# Risks and benefits of zolpidem use in Taiwan: a narrative review

**DOI:** 10.7603/s40681-016-0008-2

**Published:** 2016-05-06

**Authors:** Shih-Wei Laia

**Affiliations:** 1 College of Medicine, China Medical University, 404 Taichung, Taiwan; 2 bDepartment of Family Medicine, China Medical University Hospital, 404 Taichung, Taiwan

**Keywords:** Zolpidem, Risk, Benefit

## Abstract

Zolpidem is a non-benzodiazepine hypnotic drug commonly used for the treatment of insomnia. However, to date, extensive evidence has shown that zolpidem use is a factor associated with certain clinical conditions, not that it treats these conditions. The aim of this review is to summarize current published articles on the risks and benefits of zolpidem use.

## 1. Introduction

Zolpidem is a non-benzodiazepine hypnotic agent which is commonly used for the treatment of patients with insomnia. Although it has a few unique benefits, there is a growing body of evidence based on the database of the Taiwan National Health Insurance Program that zolpidem use could be potentially associated with certain clinical conditions. Considering how frequently zolpidem is prescribed in Taiwan [1, 2], the safety of zolpidem use is potentially a major public health issue. Therefore, we have herein reviewed and summarized recent data on zolpidem for clinicians to weigh its risks and benefits.

## 2. Clinical benefits

In the beginning, zolpidem was widely prescribed by clinicians because of its unique pharmacological profile, including sedative action at relatively low doses [3, 4], rapid onset of action [5, 6], and short elimination half-life (approximately 1.5 to 2.4 h) [6, 7]. These favorable properties allow patients who take it to rapidly fall asleep, and to increase total sleep time and decrease sleep latency [8-10]. The patients either do not feel or only slightly feel residual impaired cognition the next morning [8, 10, 11]. Overall, it has been observed that the sleep quality of patients using zolpidem really does improve.

## 3. Clinical risks

Along the aforementioned benefits, recently many studies have reported a few clinical conditions could be potentially associated with zolpidem use. What follows is a summary of these conditions.

## 4. Risks to the central nervous system

Zolpidem use has been associated with risks to the central nervous system, risks such as ischemic stroke (odds ratio = 1.37, 95% confidence interval = 1.30-1.44) [12], Parkinson’s disease (hazard ratio of incidence = 1.88, 95% confidence interval = 1.45-2.45) [13], epilepsy (odds ratio = 1.86, 95% confidence interval = 1.70-2.03) [14], benign brain tumors (hazard ratio = 1.85, 95% confidence interval = 1.21-2.82) [15], and dementia (odds ratio = 1.33, 95% confidence interval = 1.24-1.41) [16].

## 5. Risk of cancer

Zolpidem use has been associated with the risk of cancer development (hazard ratio = 1.68, 95% confidence interval 1.55-1.82) [17].

## 6. Risks of infection and inflammation

Zolpidem use has been associated with the risks of infectious events (relative risk = 2.1, *P* < 0.001) [18], acute pancreatitis (odds ratio = 7.20, 95% confidence interval = 5.81- 8.92) [19], and pyogenic liver abscesses (odds ratio = 3.89, 95% confidence interval = 2.89-5.23) [20].

## 7. Risks of injury

Zolpidem use has been associated with injuries, such as an increased risk of hospitalization related to motor vehicle accidents (odds ratio = 1.74, 95% confidence interval = 1.25-2.43) [21], an increased risk of hospitalization related to head injury or fracture (hazard ratio = 1.67, 95% confidence interval = 1.19-2.34) [22], and an increased risk of hospitalization related to hip fracture (hazard ratio of incidence = 2.28, 95% confidence interval = 1.61-3.23) [23].

## 8. Risks of other conditions

Other clinical conditions that might be potentially associated with zolpidem use are doctor shopping behavior for procurement of zolpidem [24], adverse pregnancy outcomes including low-birthweight infants (odds ratio = 1.39, 95% confidence interval = 1.17-1.64), preterm deliveries (odds ratio = 1.49, 95% confidence interval = 1.28-1.74), small-for-gestational-age infants (odds ratio = 1.34, 95% confidence interval =1.20-1.49), and cesarean delivery (odds ratio = 1.74 , 95% confidence interval = 1.59-1.90) [25], and glaucoma (odds ratio = 1.19, 95% confidence interval = 1.02-1.38) [26].

## 9. Conclusion

Despite zolpidem having its specific beneficial pharmacological effects on patients suffering from insomnia, from an overall evidence-based view using the database of the Taiwan National Health Insurance Program, there are serious potential risks to prescribing zolpidem. It is the job of clinicians, then, to consider these potential risks in addition to the established benefits of zolpidem use when considering prescribing zolpidem.
